# Characterization, Comparison of Four New Mitogenomes of Centrotinae (Hemiptera: Membracidae) and Phylogenetic Implications Supports New Synonymy

**DOI:** 10.3390/life12010061

**Published:** 2022-01-03

**Authors:** Ruitao Yu, Leining Feng, Christopher H. Dietrich, Xiangqun Yuan

**Affiliations:** 1 Key Laboratory of Plant Protection Resources and Pest Management, Ministry of Education, Entomological Museum, College of Plant Protection, Northwest A&F University, Yangling, Xianyang 712100, China; wrightyu@nwafu.edu.cn (R.Y.); fengleining@163.com (L.F.); 2 Illinois Natural History Survey, Prairie Research Institute, University of Illinois, Champaign, IL 61820, USA; chdietri@illinois.edu

**Keywords:** treehopper, Gargarini, new synonymy, mitochondrial DNA, phylogenetic analysis

## Abstract

To explore the phylogenetic relationships of the subfamily Centrotinae from the mitochondrial genome data, four complete mitogenomes (*Anchon lineatus*, *Anchon yunnanensis*, *Gargara genistae* and *Tricentrus longivalvulatus*) were sequenced and analyzed. All the newly sequenced mitogenomes contain 37 genes. Among the 13 protein-coding genes (PCGs) of the Centrotinae mitogenomes, a sliding window analysis and the ratio of Ka/Ks suggest that *atp8* is a relatively fast evolving gene, while *cox1* is the slowest. All PCGs start with ATN, except for *nad5* (start with TTG), and stop with TAA or the incomplete stop codon T, except for *nad2* and *cytb* (terminate with TAG). All tRNAs can fold into the typical cloverleaf secondary structure, except for *trnS1*, which lacks the dihydrouridine (DHU) arm. The BI and ML phylogenetic analyses of concatenated alignments of 13 mitochondrial PCGs among the major lineages produce a well-resolved framework. Phylogenetic analyses show that Membracoidea, Smiliinae and Centrotinae, together with tribes Centrotypini and Leptobelini are recovered as well-supported monophyletic groups. The tribe Gargarini (sensu Wallace et al.) and its monophyly are supported.

## 1. Introduction

Membracidae (Hemiptera: Cicadomorpha: Membracoidea) is a relatively large and widespread family within the superfamily Membracoidea, currently comprising approximately 3450 species, 428 genera and 9 subfamilies worldwide. Centrotinae, the largest and the only cosmopolitan subfamily, comprises nearly 1350 species and 216 genera. These taxa above are mainly distributed in the New World, while a buffalo treehopper species, *Stictocephala bisonia* (Kopp & Yonke, 1977), is currently widespread in Europe and Asia after being introduced by accident [1,2,3]. Some species are well-documented agricultural pests. For example, *S. bisonia* causes apple and other fruit trees to wilt by laying eggs in the twigs of those trees, *Spissistilus festinus* (Say) may infest soybeans with such large populations that ovipositional scars can impact yields, and *Metcalfiella monogramma* (Germar) may cause similar damage in avocados [4,5].

Though many phylogenetic studies of higher taxa of Membracoidea (leafhoppers and treehoppers) have been conducted [6,7,8,9,10,11], most have focused on the relationships between subfamilies or tribes within Cicadellidae, while the relationships among tribes and genera of the family Membracidae remain very poorly understood. Although the monophyly of Membracoidea (sensu lato, including Cicadellidae) has been well supported by previous analyses that sampled broadly across Membracoidea or Auchenorrhyncha [12,13], Membracidae (sensu Deitz & Dietrich, 1993) has not been consistently recovered as monophyletic in some recent analyses [9,10,14,15]. Some analyses have also suggested that the largest membracid subfamily, Centrotinae, is paraphyletic [9,10,14,16]. The only detailed phylogenetic analysis of relationships within Centrotinae was based on morphology [3] and their hypothesis has not yet been tested by incorporating molecular data. The recent anchored hybrid-based phylogenomic analysis of Membracoidea [12] recovered Centrotinae as a monophyletic group with strong support and suggested that Centrotinae arose in the New World and later colonized the Old World. Further analyses incorporating more taxa are needed to provide a more robust estimate of relationships among tribes within Centrotinae.

Due to the innovation of next-generation sequencing (NGS), the mitogenome has become an important molecular indicator in the study of insect systematics and has been widely used in phylogenetic studies of different taxa levels with various insects because of its maternal inheritance, compositional stability and genetic conservation [17,18,19]. Although some studies have indicated that the mitogenome itself is insufficient to resolve the higher-level phylogeny of Auchenorrhyncha [20,21,22], analyses based on the complete sequence of mitochondrial DNA may still help to resolve the ongoing controversies in the classification and the phylogenetic relationships of the Centrotinae.

## 2. Materials and Methods

### 2.1. Specimen Acquisition

The detailed species list of the adult Centrotinae used in the study is shown in Table 1. All the treehoppers were immersed in 100% ethyl ethanol after capture and stored at a −20 degrees Celsius freezer to preserve the DNA, and the specimens were identified based on morphological taxonomic characters [23]. All experimental insects were preserved at the Entomological Museum of Northwest A&F University.

### 2.2. DNA Extraction, Mitogenome Sequencing, Assembly and Annotation

For sequencing mitogenomes, we used DNeasy DNA Extraction Kit (Qiagen) to extract the total genomic DNA from thoracic muscle tissues. The NGS (Illumina HiSeq X; Biomarker Tech, Beijing, China) was employed to determine the four mitogenomes of Centrotinae. A total of 16,902,362/13,815,488/20,016,944/13,564,230 clean paired reads, then assembled using Geneious 9.0.2 [24] with the mitogenomes of *Leptobelus gazella* (JF801955) and *Tricentrus brunneus* (MK746138) were employed as references. The annotation of the mitogenomes was performed using Geneious 9.0.2. Furthermore, the MITOS Web Server (Leipzig, Germany) [25], with the invertebrate mitochondrial genetic code (transl_table = 5), was made a forecast for the position and secondary structure of the tRNA, and Adobe Illustrator 2021 was employed to draw manually as the predicted results show. The PCGs boundaries were recognized by the open reading frames (ORFs) employing translation table 5 and alignment with homologous reference sequences was performed in Geneious 9.0.2. In addition, CGView Server (http://cgview.ca/ (accessed on 26 June 2021)) [26] was used to generate the mitogenome maps online.

### 2.3. Bioinformatic Analysis

The base composition and relative synonymous codon usage (RSCU) values were computed using RStudio Desktop 1.4.1106 [27] and PhyloSuite v1.2.2 [28]. DnaSP v6 [29] was utilized to conduct the sliding window analysis (a sliding window of 200 bp and step size of 20 bp) and calculate the nucleotide diversity (Pi value) and the ratio of non-synonymous substitution rate (Ka) to synonymous substitution rate (Ks) of aligned PCGs. Genetic distances based on the PCGs were estimated employing MEGA X [30] with Kimura 2-parameter. Prism 9.0.0 was used to plot graphically the genetic distances and Ka/Ks ratios. The Centrotinae species (*A. lineatus*, *A. yunnanensis*, *G. genistae* and *T. longivalvulatus*) mitogenome sequences were uploaded on GenBank with accession numbers MZ504904, MZ504905, MZ504906, and MZ504907, respectively (Table 2).

### 2.4. Phylogenetic Analysis

For phylogenetic analysis, 55 species of Membracoidea (42 leafhoppers and 13 treehoppers) representing 12 subfamilies in 3 families were considered as ingroups. Outgroups are four representative species from four families in two different superfamilies: *Philaenus spumarius* (Cercopoidea: Aphrophoridae: Aphrophorinae), *Callitettix braconoides* (Cercopoidea: Cercopidae: Callitettixinae), *Magicicada tredecula* (Cicadoidea: Cicadidae: Cicadettinae) and *Tettigarcta crinita* (Cicadoidea: Tettigarctidae: Tettigarctinae). All species sequences are available on GenBank (Table 2).

PhyloSuite v1.2.2 was employed to extract the genes. Alignments of all 13 PCGs and 2 rRNA genes were based on Q-INS-i strategy and G-INS-i strategy, respectively, using the MAFFT v7.313 plugin [31] in PhyloSuite. Gblocks 0.91b [32] was used to remove poorly aligned regions. Moreover, MEGA X was used to check and correct all alignments manually. Then, all correctly aligned gene sequences of each species were concatenated.

Based on the PCG123 dataset (all codon positions of the 13 PCGs), phylogenetic reconstruction was performed. The best-fit partitioning strategies were determined by PartitionFinder 2 plugin integrated into PhyloSuite [33] employing the “greedy” algorithm and Bayesian information criterion (BIC) (shown in Tables S1 and S2). IQ-TREE v.1.6.8 was employed to perform a maximum likelihood (ML) analysis [34]. Bootstrap support (BS) was assessed under 1000 ultrafast bootstraps (UFB) replicates [35]. Bayesian inference (BI) analysis was performed using MrBayes v3.2.6 [36] with default settings and Markov chain Monte Carlo (MCMC) runs were performed for 5 × 10^6^ generations sampling every 1000 generations, with the first 25% discarded as burn-in, as implemented in the CIPRES Science Gateway [37].

**Table 2:** The mitogenomic sequences used in this study.

Superfamily	Family/Subfamily	Species	Accession Number	Reference
Outgroup				
Cercopoidea	Aphrophoridae/Aphrophorinae	*Philaenus spumarius*	NC_005944	[38]
	Cercopidae/Callitettixinae	*Callitettix braconoides*	NC_025497	[39]
Cicadoidea	Cicadidae/Cicadettinae	*Magicicada tredecula*	MH937705	[40]
	Tettigarctidae/Tettigarctinae	*Tettigarcta crinita*	MG737758	[41]
Ingroup				
Membracoidea	Cicadellidae/Cicadellinae	*Bothrogonia ferruginea*	KU167550	Unpublished
		*Cicadella viridis*	KY752061	Unpublished
		*Homalodisca vitripennis*	NC_006899	Unpublished
		*Olidiana ritcheriina*	MK738125	Unpublished
		*Taharana fasciana*	NC_036015	[42]
	Cicadellidae/Deltocephalinae	*Drabescoides nuchalis*	NC_028154	[43]
		*Japananus hyalinus*	NC_036298	[44]
		*Macrosteles quadrilineatus*	NC_034781	[45]
		*Macrosteles quadrimaculatus*	NC_039560	[46]
		*Maiestas dorsalis*	NC_036296	[44]
		*Nephotettix cincticeps*	NC_026977	Unpublished
		*Scaphoideus maai*	KY817243	[47]
		*Scaphoideus nigrivalveus*	KY817244	[47]
		*Scaphoideus varius*	KY817245	[47]
		*Tambocerus* sp.	KT827824	[48]
		*Yanocephalus yanonis*	NC_036131	[47]
	Cicadellidae/Evacanthinae	*Evacanthus acuminatus*	MK948205	[49]
		*Evacanthus heimianus*	MG813486	[50]
	Cicadellidae/Iassinae	*Batracomorphus lateprocessus*	MG813489	[51]
		*Gessius rufidorsus*	MN577633	[51]
		*Krisna concava*	MN577635	[51]
		*Krisna rufimarginata*	NC_046068	[51]
		*Trocnadella arisana*	NC_036480	[51]
	Cicadellidae/Idiocerinae	*Idiocerus laurifoliae*	NC_039741	[52]
		*Idioscopus clypealis*	NC_039642	[53]
		*Idioscopus myrica*	MH492317	[52]
		*Idioscopus nitidulus*	NC_029203	[54]
		*Populicerus populi*	NC_039427	[52]
	Cicadellidae/Ledrinae	*Ledra auditura*	MK387845	[55]
		*Petalocephala chlorophana*	MT610899	[56]
		*Tituria pyramidata*	MN920440	Unpublished
		*Tituria sagittata*	MT610900	[56]
	Cicadellidae/Megophthalminae	*Durgades nigropicta*	NC_035684	[57]
		*Japanagallia spinosa*	NC_035685	[57]
	Cicadellidae/Typhlocybinae	*Bolanusoides shaanxiensis*	MN661136	Unpublished
		*Empoascanara dwalata*	MT350235	Unpublished
		*Empoasca onukii*	NC_037210	[58]
		*Empoascanara sipra*	MN604278	[59]
		*Ghauriana sinensis*	MN699874	[60]
		*Limassolla lingchuanensis*	NC_046037	[61]
		*Mitjaevia protuberanta*	NC_047465	[62]
		*Paraahimia luodianensis*	NC_047464	[63]
	Aetalionidae/Aetalioninae	*Darthula hardwickii*	NC_026699	[64]
	Membracidae/Smiliinae	*Entylia carinata*	NC_033539	[65]
		*Stictophala bisonia*	MW342606	[66]
	Membracidae/Centrotinae	*Anchon lineatus*	MZ504904	This study
		*Anchon yunnanensis*	MZ504905	This study
		*Gargara genistae*	MZ504906	This study
		*Hypsauchenia hardwichii*	MK746135	[2]
		*Leptobelus gazella*	JF801955	[67]
		*Leptobelus* sp.	JQ910984	[68]
		*Leptocentrus albolineatus*	MK746137	[2]
		*Maurya qinlingensis*	MK746136	[2]
		*Tricentrus longivalvulatus*	MZ504907	This study
		*Tricentrus brunneus*	MK746138	[2]

## 3. Results

### 3.1. Genome Organization and Base Composition

**Figure 1:**
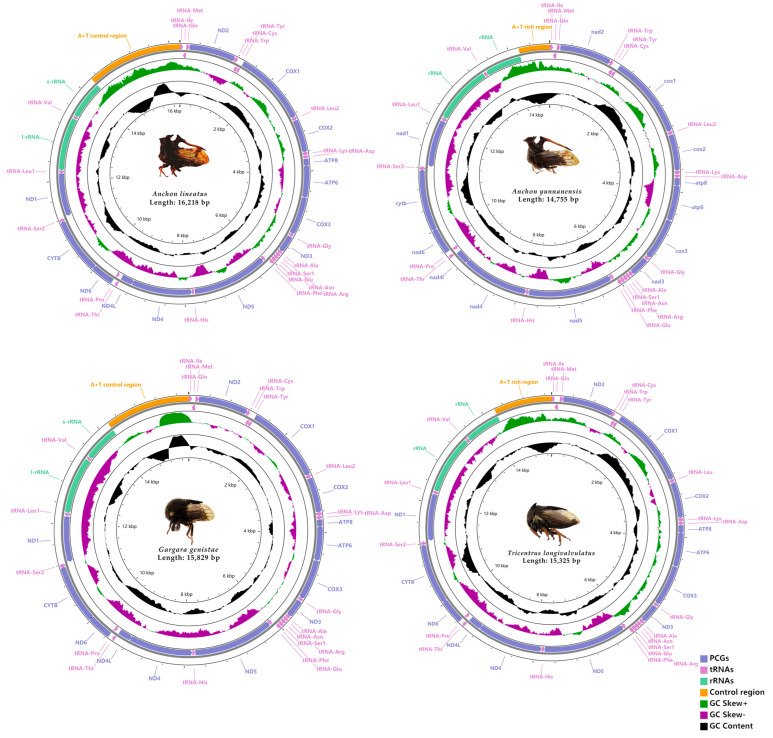
Circular maps of the mitogenomes of A. lineatus, A. yunnanensis, G. genistae and T. longivalvulatus.

The newly sequenced mitogenomes of *A. lineatus*, *A. yunnanensis*, *G. genistae* and *T. longivalvulatus* were all double-stranded, circular molecules, with the total lengths of 16,218 bp, 14,775 bp, 15,829 bp and 15,325 bp, respectively (Figure 1). Among the 4 complete mitogenomes of Centrotinae, *A. yunnanensis* had the smallest mitogenome at 14,775 bp, while *A. lineatus* had the largest at 16,218 bp. Variation in the length of mitogenomes is primarily caused by the variable non-coding region. All mitogenomes included the 37 typical invertebrate mitochondrial genes (13 PCGs, 22 tRNA genes and 2 rRNA genes) and all the genes were identified (Figure 1). There were 23 genes on the majority strand (J-strand), whereas 14 genes were located on the minority strand (N-strand) (Tables S4–S7). The gene order and organization of the four newly determined Centrotinae have high consistency compared with the typical previously reported membracid species. The base composition of total genome of *A. lineatus* is A (45.3%), T (31.1%), C (14.4%) and G (9.2%); *A. yunnanensis* is A (45.3%), T (31.0%), C (14.5%) and G (9.2%); *G. genistae* is A (43.0%), T (34.0%), C (12.0%) and G (10.9%); and A (44.2%), T (32.7%), C (13.6%) and G (9.6%) in *T. longivalvulatus* (see Table S3). Similar to other Membracidae mitogenomes, the four mitogenomes are highly AT biased, with 76.4% in *A. lineatus*, 76.3% in *A. yunnanensis*, 77.0% in *G. genistae*, and 76.9% in *T. longivalvulatus*. All mitogenomes show a strong AT bias and a positive AT-skew and CG-skew (Table S3). 

### 3.2. Protein-Coding Genes and Codon Usage

The total lengths of the 13 PCGs of *A. lineatus*, *A. yunnanensis*, *G. genistae* and *T. longivalvulatus* are 10,908 bp, 10,902 bp, 10,920 bp and 10,911 bp, respectively (Table S3). In the 4 newly sequenced mitogenomes, 9 of the 13 PCGs are located on the J-strand and others are on the N-strand. The AT-skews are −0.12, −0.117, −0.14, and −0.148, respectively (Table S3). Except for *nad5* in *A. yunnanensis* and *T. longivalvulatus* (using TTG as start codon), most PCGs start with ATN as in the previously reported Centrotinae *Hypsauchenia hardwickii*, *Maurya qinlingensis* (MK746136), *Tricentrus brunneus* (MK746138), *Leptocentrus albolineatus* (MK746137) and *Leptobelus* sp. HL-2012 (JQ910984). The typical codon TAA and incomplete single T (mostly occurring on *cox1*, *cox2*, *nad5*) were used as the stop codon. It is worth mentioning that *nad2* and *cytb* in *A. lineatus*, *A. yunnanensis* and *G. genistae* use TAG as a termination. The stop codon TAA is used more frequently than TAG, and three single Ts are present at least in all four Centrotinae mitogenomes (Tables S4–S7). Such incomplete termination codons occur universally in insect mitogenomes; they are thought to be completed by post-transcriptional polyadenylation modification during mRNA maturation.

**Figure 2:**
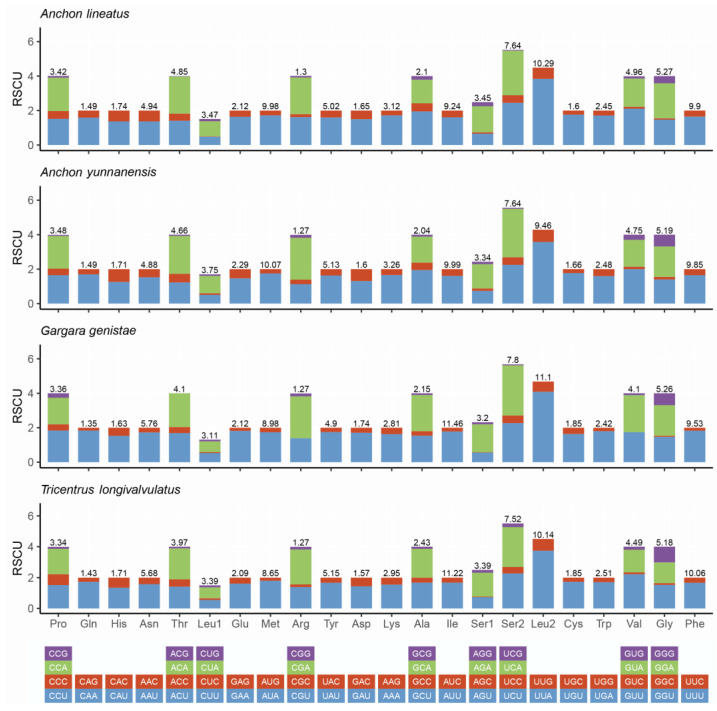
Relative synonymous codon usage (RSCU) of the mitogenomes of four Centrotinae species.

The RSCU values and the amino acid compositions are shown in Figure 2. AUU (Ile), UUA (Leu2), UUU (Phe) and AUA (Met) are the most frequently used codons with only component A or U. The third codon is biased toward A or T (Figure 2), which shows the A + T bias of the protein-coding genes in mitogenomes among Centrotinae. 

### 3.3. Transfer and Ribosomal RNA Genes

The 22 transfer RNA genes (tRNAs) of each species discontinuously appeared over the whole mitogenome (Tables S4–S7). The lengths of the tRNA regions of these mitogenomes are similar with 1413 bp in *A. lineatus*, 1415 bp in *A. yunnanensis*, 1402 bp in *G. genistae* and 1393 bp in *T. longivalvulatus*. The AT content (ranging from 78.6% to 79.8%) of the tRNA is moderately higher than that (ranging from 74.8% to 77.0%) of the PCGs (Table S3). The positions of the 22 tRNAs are identified in the same relative genomic positions as previously determined Membracidae. The lengths of these 22 tRNA genes range from 60 bp (*trnG*) to 70 bp (*trnK*) in *A. lineatus*, from 61 bp (*trnG*, *trnH*, and *trnT*) to 70 bp (*trnK*) in *A. yunnanensis*, from 59 bp (*trnT*) to 71 bp (*trnK*) in *G. genistae*, and from 60 bp (*trnC* and *trnR*) to 71 bp (*trnK*) in *T. longivalvulatus*. As presented in Figures S1–S4, all tRNAs exhibit typical clover-leaf secondary structure, but *trnS1* (AGN) lacks the dihydrouridine (DHU) arm, as identified in other membracoid species. The phenomenon of lacking is also generally found in metazoan mitochondrial genomes [69]. There are eight mismatched types (G–U, U–U, A–A, A–C, A–G, G–G, single U and single A) of incorrectly paired bases in these four mitogenomes. A total of 25 weak-bonded G–U, 7 mismatched U–U, 5 mismatched A–A, 2 mismatched A–C, 1 mismatched A–G and 1 mismatched G–G are found in *A. lineatus*. A total of 25 weak-bonded G–U, 11 mismatched U–U, 5 mismatched A–A, 1 mismatched A–C, and 1 mismatched A–G are found in *A. yunnanensis*. Furthermore, 31 weak-bonded G–U, 11 mismatched U–U, 2 mismatched A–A, 1 mismatched A–G, 1 single A and U are found in *G. genistae*, and 25 weak-bonded G–U, 12 mismatched U–U, 3 mismatched A–C, 1 mismatched A–G, and 1 mismatched A–A are discovered in *T. longivalvulatus*. 

In the four newly sequenced mitogenomes, two rRNA genes (*rrnL* and *rrnS*) were found to be encoded on the N-strand. The *rrnLs* are 1162/1162/1171/1154 bp (*A. lineatus*/*A. yunnanensis*/*G. genistae*/*T. longivalvulatus,* respectively) in size, located between *trnL1* (CUN) and *trnV*, while the *rrnSs* are 824/736/739/736 bp (*A. lineatus*/*A. yunnanensis*/*G. genistae*/*T. longivalvulatus,* respectively) in size and reside between *trnV* and control region (Tables S4–S7). The two genes have a negative AT skew (ranging from −0.220 to −0.189) and positive GC skew (ranging from 0.240 to 0.276) in these four mitogenomes (Table S3).

### 3.4. Gene Overlaps

A total of 17/16/16/11 gene overlaps occur in the *A. lineatus*/*A. yunnanensis*/*G. genistae*/*T. longivalvulatus* mitogenomes, respectively, with sizes from 1 bp to 14 bp. The largest overlap found of the four mitogenomes is 14 bp, between *nad6* and *cytb,* occurring in *G. genistae* (Tables S4–S7). One identical overlap in *nad6*-*cytb* (ATGAATAA) is found in all four Centrotinae species. There are 6/4/8/9 intergenic spacers in the four mitogenomes, respectively, ranging from 1 bp to 27 bp and the longest intergenic spacer is between *trnQ* and *trnM* in *T. longivalvulatus* (Tables S4–S7). None of the newly sequenced mitogenomes share an identical intergenic spacer.

### 3.5. Non-Coding Regions

The control region is considered as the longest non-coding region in the sequenced mitogenomes. The lengths are 1940 bp in *A. lineatus*, 570 bp in *A. yunnanensis*, 1633 bp in *G. genistae*, and 1099 bp in *T. longivalvulatus* (Table S3). The A + T contents are 79.8% in *A. lineatus*, 90.7% in *A. yunnanensis*, 71.7% in *G. genistae* and 83.8% in *T. longivalvulatus*. 

### 3.6. Nucleotide Diversity and Evolutionary Rate Analysis

**Figure 3:**
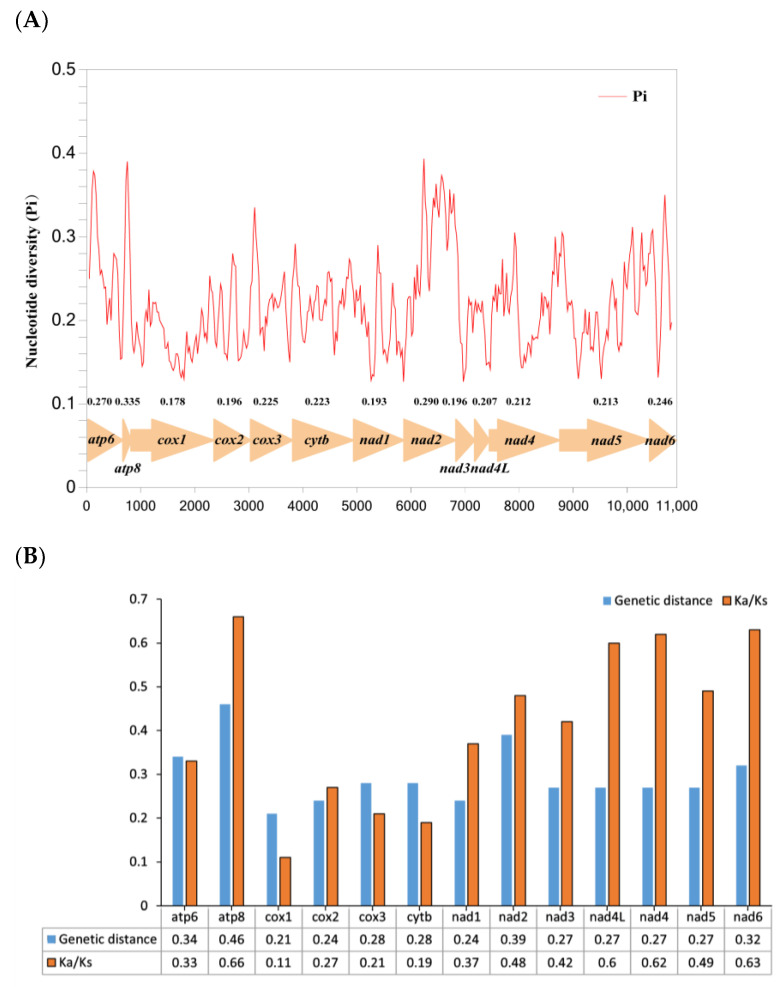
Nucleotide diversity (Pi) (**A**) and genetic distance and ratio of non-synonymous (Ka) to synonymous (Ks) substitution rates (**B**) of PCGs from four Centrotinae mitogenomes.

Nucleotide diversity of the 13 PCGs by sliding window analysis is shown in Figure 3A. Genes *atp8*, *nad2*, *atp6*, and *nad6* have relatively high nucleotide diversities of 0.335, 0.290, 0.270, and 0.246, respectively, while genes *cox1*, *nad1*, *cox2*, and *nad3* have comparatively low nucleotide diversities of 0.178, 0.193, 0.196, and 0.196, respectively. Pairwise genetic distance analysis also presents similar results with high distances of 0.46, 0.39, 0.34, and 0.32 for *atp8*, *nad2*, *atp6*, and *nad6*, respectively, and low distances of 0.21, 0.24, 0.24, and 0.27 for *cox1*, *nad1*, *cox2*, and *nad3*, separately (Figure 3B). 

The ratio of Ka/Ks (ω) was calculated to further analyze the evolutionary rate of 13 protein coding genes. The Ka/Ks values range from 0.11 to 0.66, implying that PCGs are evolving under a purifying selection. Genes, such as *atp8*, *nad6*, *nad4* and *nad4L*, show relatively high Ka/Ks ratios of 0.66, 0.63, 0.62 and 0.6, respectively, suggesting that they have undergone comparatively weak purifying pressure, while *cox1*, *cytb*, *cox3* and *cox2* demonstrate fairly low values of 0.11, 0.19, 0.21 and 0.27, separately, which shows these genes are likely to be under the strongest purifying selection (Figure 3B).

### 3.7. Phylogenetic Relationships

**Figure 4:**
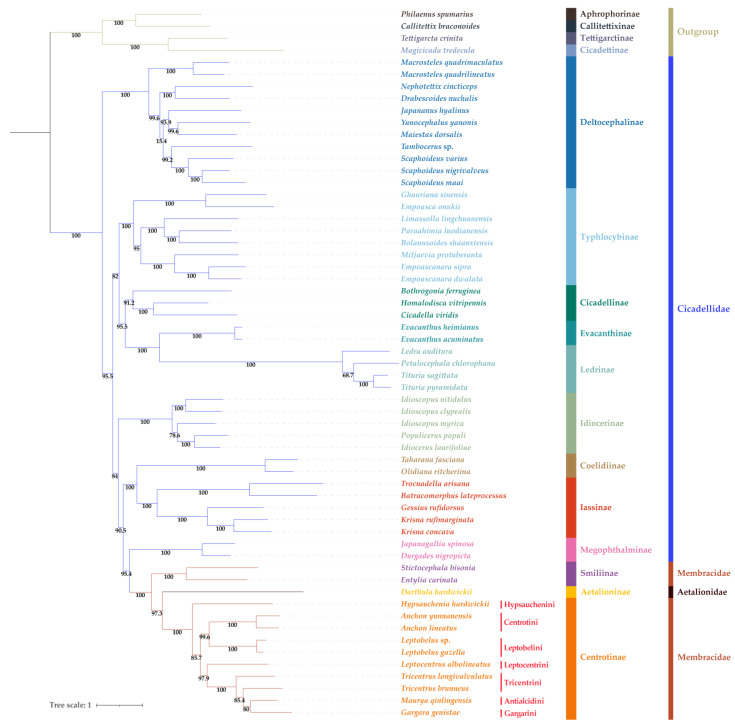
ML tree inferred from IQ-TREE analyses. Numbers on nodes are the bootstrap support values (BS).

**Figure 5:**
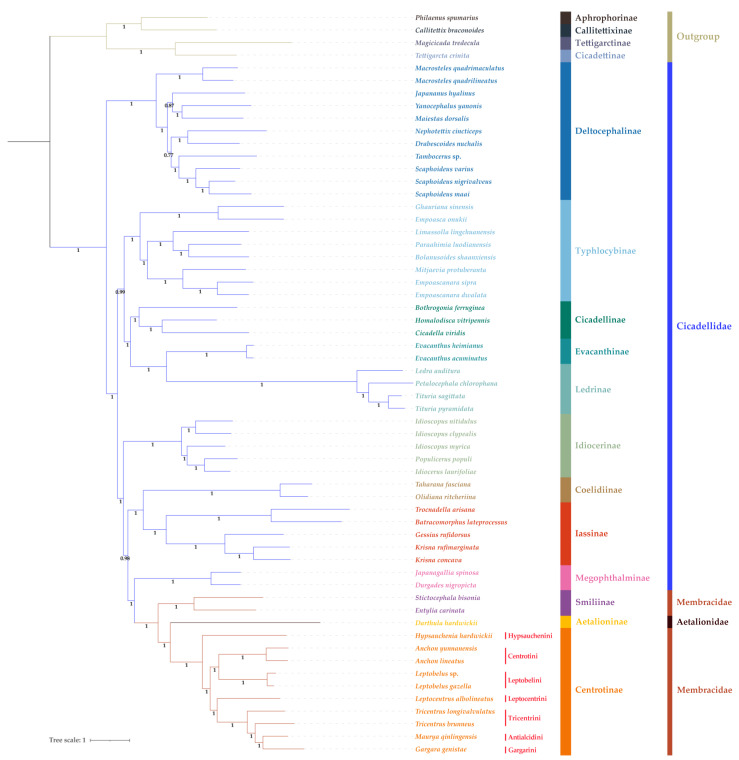
BI tree inferred from MrBayes analyses. Numbers on nodes are the Bayesian posterior probabilities (PP).

The phylogenetic analyses of 59 species of Cercopoidea, Cicadoidea, and Membracoidea inferred based on ML and BI analyses of the PCG123 dataset yielded highly congruent topologies, with most branches receiving strong support (Figure 4 and Figure 5). The monophyly of Membracoidea was recovered (BS = 100, PP = 1). Membracoidea was divided into two major clades. One clade composed of the eleven Deltocephalinae species formed a sister group to a second group comprised of the remaining leafhoppers and all of Membracidae with strong support (BS = 100; PP = 1) received both in the ML tree and BI tree. Within Membracoidea, as in other recent phylogenetic studies, some early divergences within Cicadellidae, pertaining to relationships among subfamilies and tribes, are not well resolved. Specifically, the relationships among tribes in Deltocephalinae, especially Opsiini, Paralimnini, Deltocephalini, Chiasmini, and Drabescini, remain unstable. Relationships among Typhlocybinae, Cicadellinae, Evacanthinae, Ledrinae, Idiocerinae, Coelidiinae, Iassinae, Megophthalminae, Smiliinae, Aetalioninae and Centrotinae are congruent in both the ML tree and the BI tree (Figure 4 and Figure 5). Treehoppers (Membracidae and Aetalionidae) are a monophyletic group sister to Megophthalminae and derived from a paraphyletic lineage of leafhoppers (Cicadellidae).

## 4. Discussion

In this study, the sequenced-mitogenome genes of these Centrotinae species are found to be highly conserved, similar to other Membracidae. Phylogenetic analyses indicate that Membracoidea was divided into two major clades is consistent with several previous studies [9,38,40,41]. The relationships among tribes in Deltocephalinae, especially Opsiini, Paralimnini, Deltocephalini, Chiasmini, and Drabescini, remain unstable. Previous analyses have also yielded inconsistent results for this group [70,71]. The Membracidae subfamilies Smiliinae and Centrotinae are both recovered as monophyletic groups, which is generally consistent with previous studies [8,9,10,40,65,66], but Membracidae itself is paraphyletic with respect to Aetalionidae. To better understand the relationships within the treehopper lineage, data for representatives of additional subfamilies are needed because only two subfamilies are represented in the current dataset. A relationship Smiliinae + (Aetalionidae + Centrotinae) within treehoppers in both phylogenetic topologies has been recovered, which is congruent with some previous research [2,40], but, given the lack of data for other New World subfamilies (i.e., Centronodinae, Darninae, Heteronotinae, Membracinae and Nicomiinae), our results should be interpreted with caution. Within Centrotinae, the largest membracid subfamily, our results (Figure 4 and Figure 5) support the monophyly of Centrotini and Leptobelini, but the monophyly of Tricentrini is rejected according to the classification system of Membracoidea from China proposed by Yuan and Chou [23]. However, the tribes Tricentrini, Gargarini, and Antialcidini share many morphological characters, for example, they possess posterior process on pronotum and no dentatus on either side of the mesonotum; three apical cells on hindwings; and the scutellum is covered by a posterior process, and only two sides are exposed [23]. Moreover, according to the revised classification proposed by Wallace et al. [3], Tricentrini Ahmad et Yasmeen, 1974 (new **Synonym**), Gargarini Distant, 1907 sensu stricto, and Antialcidini Yuan et Zhang, 2002 (new **Synonym** nova) are all included in Gargarini sensu lato. In consideration of these, our analyses recover Gargarini (sensu Wallace et al.) as a monophyletic group. Relationships among the included tribes within Centrotinae can be inferred as (Hypsaucheniini + ((Centrotini + Leptobelini) + (Leptocentrini + Gargarini))). 

## 5. Conclusions

The complete mitogenomes of *A. lineatus, A. yunnanensis, G. genistae and T. longivalvulatus* are newly sequenced in this study, and the structural characteristics and nucleotide compositions are found to be similar to those of other Membracoidea species as well as to the hypothetical ancestral insect mitogenome. The BI and ML phylogenetic analyses of concatenated alignments of 13 mitochondrial PCGs among the major lineages yield well-resolved topologies, with most branches receiving moderate to strong support. Membracoidea, membracid subfamilies Smiliinae, Centrotinae, and tribes within Centrotinae, Centrotini and Leptobelini are recovered as well-supported monophyletic groups, while Tricentrini, in the traditional sense, is paraphyletic. The tribe Gargarini (sensu Wallace et al.) is supported and recovered as a monophyletic group. At a tribe level, the relationship [Hypsaucheniini + ((Centrotini + Leptobelini) + (Leptocentrini + Gargarini))] is recovered based on ML and BI analyses. 

Taken together, mitogenomic data are helpful in reconstructing the phylogenetic relationships of Membracoidea, at least at the subfamily and tribe levels, consistent with previous analyses of other kinds of data. However, knowledge of phylogenetic relationships within this group continues to be hindered by the extremely limited number of taxa that have, so far, been included in molecular datasets, including those based on complete mitogenomes. Based on our results, further sequencing of mitogenomes is expected to yield increasingly robust estimates of relationships among major lineages of Membracoidea.

## Figures and Tables

**Table 1:** Collection information of the Centrotinae species sequenced this study.

Organism	Locality	Time	Collector
*Anchon lineatus*	Jinghong, Yunnan	8 July 2017	Hu-Kai
*Anchon yunnanensis*	Jinghong, Yunnan	9 July 2017	Hu-Kai
*Gargara genistae*	Northwest A&F University, Yangling, Shaanxi	13 June 2018	Hu-Kai
*Tricentrus longivalvulatus*	Ruyuan, Guangdong	24 July 2020	Yu-Ruitao

## Data Availability

The data that support the findings of this study are openly available in GenBank of NCBI at https://www.ncbi.nlm.nih.gov (accessed on 5 August 2021), reference number MZ504904, MZ504905, MZ504906, and MZ504907.

## Supplementary Materials

The following are available online at https://www.mdpi.com/article/10.3390/life12010061/s1: Figure S1: Secondary structure for the tRNA genes of *Anchon lineatus*; Figure S2: Secondary structure for the tRNA genes of *Anchon yunnanensis*; Figure S3: Secondary structure for the tRNA genes of *Gargara genistae*; Figure S4: Secondary structure for the tRNA genes of *Tricentrus longivalvulatus*; Table S1: The best partitioning schemes and substitution models for PCG123 dataset comprising 13 PCGs of 59 species used for ML phylogenetic analyses; Table S2: The best partitioning schemes and substitution models for PCG123 dataset comprising 13 PCGs of 59 species used for BI phylogenetic analyses; Table S3: Nucleotide composition and skewness comparison of the four mitogenomes; Table S4: Structural organization of the mitogenome of *A. lineatus*; Table S5: Structural organization of the mitogenome of *A. yunnanensis*; Table S6: Structural organization of the mitogenome of *G. genistae*; Table S7: Structural organization of the mitogenome of *T. longivalvulatus.*
