# Supplementary material for: Characterization, Comparison of Four New Mitogenomes of Centrotinae (Hemiptera: Membracidae) and Phylogenetic Implications Supports New Synonymy
Source: Life (Basel). 2022 Jan 3;12(1):61. doi: 10.3390/life12010061 (PMC8777817; doi:10.3390/life12010061)
Supplement: Supplementary file 1 [file life-12-00061-s001.zip › Supplementary Table S1.pdf]

Table S1. The best partitioning schemes and substitution models for PCG123 dataset comprising 13 PCGs of 59 species used for ML phylogenetic analyses.

**Subset**	**Best Model**	**Partition names**
Partition1	GTR+I+G	*nad4L*_codon1, *atp6*_codon1, *nad1*_codon1, *nad5*_codon1, *nad4*_codon1
Partition2	TVM+I+G	*cox3*_codon2, *cytb*_codon2, *cox2*_codon2, *atp6*_codon2
Partition3	HKY+I+G	*atp6*_codon3, *cox3*_codon3, *cox2*_codon3
Partition4	GTR+I+G	*atp8*_codon1, *nad2*_codon1, *nad3*_codon1, *nad6*_codon1
Partition5	TVM+I+G	*nad3*_codon2, *nad6*_codon2, *nad2*_codon2, *atp8*_codon2
Partition6	GTR+G	*atp8*_codon3, *cytb*_codon3, *nad3*_codon3, *nad6*_codon3
Partition7	GTR+I+G	*cox1*_codon1
Partition8	TVM+I+G	*cox1*_codon2
Partition9	TIM+I+G	*cox1*_codon3
Partition10	GTR+I+G	*cox3*_codon1, *cox2*_codon1, *cytb*_codon1
Partition11	GTR+I+G	*nad4*_codon2, *nad5*_codon2, *nad1*_codon2, *nad4L*_codon2
Partition12	HKY+G	*nad1*_codon3
Partition13	TRN+G	*nad2*_codon3
Partition14	HKY+G	*nad4*_codon3, *nad4L*_codon3, *nad5*_codon3
