# Supplementary material for: Characterization, Comparison of Four New Mitogenomes of Centrotinae (Hemiptera: Membracidae) and Phylogenetic Implications Supports New Synonymy
Source: Life (Basel). 2022 Jan 3;12(1):61. doi: 10.3390/life12010061 (PMC8777817; doi:10.3390/life12010061)
Supplement: Supplementary file 1 [file life-12-00061-s001.zip › Supplementary Table S2.pdf]

Table S2. The best partitioning schemes and substitution models for PCG123 dataset comprising 13 PCGs of 59 species used for BI phylogenetic analyses.

Subset	Best Model	Partition names
Partition1	GTR+I+G	*atp6*_codon1, *nad4L*_codon1, *nad1*_codon1, *nad4*_codon1, *nad5*_codon1
Partition2	GTR+I+G	*nad3*_codon2, *atp6*_codon2, *atp8*_codon2, *nad2*_codon2, *nad6*_codon2
Partition3	GTR+I+G	*cytb*_codon3, *nad3*_codon3, *atp8*_codon3, *atp6*_codon3, *nad6*_codon3
Partition4	GTR+I+G	*atp8*_codon1, *nad2*_codon1, *nad3*_codon1, *nad6*_codon1
Partition5	GTR+I+G	*cox1*_codon1
Partition6	GTR+I+G	*cox1*_codon2, *cox3*_codon2, *cox2*_codon2, *cytb*_codon2
Partition7	HKY+I+G	*cox1*_codon3
Partition8	GTR+I+G	*cox3*_codon1, *cox2*_codon1, *cytb*_codon1
Partition9	HKY+G	*cox3*_codon3, *cox2*_codon3
Partition10	GTR+I+G	*nad5*_codon2, *nad4*_codon2, *nad1*_codon2, *nad4L*_codon2,
Partition11	HKY+G	*nad1*_codon3
Partition12	GTR+G	*nad2*_codon3
Partition13	HKY+G	*nad4*_codon3, *nad4L*_codon3, *nad5*_codon3
