# Supplementary material for: Characterization, Comparison of Four New Mitogenomes of Centrotinae (Hemiptera: Membracidae) and Phylogenetic Implications Supports New Synonymy
Source: Life (Basel). 2022 Jan 3;12(1):61. doi: 10.3390/life12010061 (PMC8777817; doi:10.3390/life12010061)
Supplement: Supplementary file 1 [file life-12-00061-s001.zip › Supplementary Table S3.pdf]

Table S3. Nucleotide composition and skewness comparison of different elements of the four mitogenomes.

Regions	Species	Size (bp)	A%	T%	C%	G%	AT(%)	GC(%)	AT skew	GC skew
PCGs	*A. lineatus*	*10,908*	*33.0*	*42.0*	*12.9*	*12.1*	*75.0*	*25.0*	*-0.120*	*-0.035*
	A. yunnanensis	*10,902*	*33.0*	*41.8*	*13.0*	*12.1*	*74.8*	*25.1*	*-0.117*	*-0.035*
	G. genistae	*10,920*	*33.1*	*43.9*	*11.4*	*11.6*	*77.0*	*23.0*	*-0.140*	*0.010*
	T. longivalvulatus	*10,911*	*32.0*	*43.2*	*12.5*	*12.3*	*75.2*	*24.8*	*-0.148*	*-0.009*
1st codon position	*A. lineatus*	*3,636*	*35.5*	*37.1*	*11.4*	*16.1*	*72.6*	*27.5*	*-0.022*	*0.170*
	A. yunnanensis	*3,634*	*36.1*	*36.4*	*11.7*	*15.8*	*72.5*	*27.5*	*-0.004*	*0.151*
	G. genistae	*3,640*	*36.2*	*37.8*	*10.7*	*15.3*	*74.0*	*26.0*	*-0.021*	*0.178*
	T. longivalvulatus	*3,637*	*35.8*	*37.4*	*11.1*	*15.7*	*73.2*	*26.8*	*-0.022*	*0.172*
2nd codon position	*A. lineatus*	*3,636*	*20.3*	*47.7*	*18.0*	*14.0*	*68.0*	*32.0*	*-0.403*	*-0.123*
	A. yunnanensis	*3,634*	*20.6*	*47.7*	*17.8*	*13.9*	*68.3*	*31.7*	*-0.398*	*-0.123*
	G. genistae	*3,640*	*20.5*	*48.2*	*17.4*	*14.0*	*68.7*	*31.4*	*-0.402*	*-0.109*
	T. longivalvulatus	*3,637*	*20.8*	*47.8*	*17.2*	*14.2*	*68.6*	*31.4*	*-0.394*	*-0.097*
3rd codon position	*A. lineatus*	*3,636*	*43.3*	*41.3*	*9.4*	*6.1*	*84.6*	*15.5*	*0.024*	*-0.215*
	A. yunnanensis	*3,634*	*42.4*	*41.2*	*9.7*	*6.7*	*83.6*	*16.4*	*0.013*	*-0.180*
	G. genistae	*3,640*	*42.7*	*45.7*	*6.1*	*5.5*	*88.4*	*11.6*	*-0.035*	*-0.045*
	T. longivalvulatus	*3,637*	*39.6*	*44.3*	*9.2*	*7.0*	*83.9*	*16.2*	*-0.056*	*-0.138*
Control region	*A. lineatus*	*1,940*	*40.5*	*39.3*	*10.1*	*10.2*	*79.8*	*20.3*	*0.003*	*0*
	A. yunnanensis	*570*	*44.4*	*46.3*	*4.7*	*4.6*	*90.7*	*9.3*	*-0.019*	*0*
	G. genistae	*1,633*	*37.5*	*34.2*	*5.9*	*22.4*	*71.7*	*28.3*	*0.584*	*0*
	T. longivalvulatus	*1,099*	*44.5*	*39.3*	*7.2*	*9.0*	*83.8*	*16.2*	*0.112*	*0*
tRNAs	*A. lineatus*	*1,413*	*40.3*	*38.7*	*8.6*	*12.3*	*79.0*	*20.9*	*0.021*	*0.176*
	A. yunnanensis	*1,415*	*39.4*	*39.2*	*9.1*	*12.4*	*78.6*	*21.5*	*0.003*	*0.151*
	G. genistae	*1,402*	*39.9*	*39.9*	*7.9*	*12.2*	*79.8*	*20.1*	*0.000*	*0.213*
	T. longivalvulatus	*1,393*	*40.0*	*38.9*	*8.9*	*12.2*	*78.9*	*21.1*	*0.014*	*0.156*

Table S3. *Cont.*

rRNAs	*A. lineatus*	*1,986*	*30.7*	*48.0*	*7.8*	*13.4*	*78.7*	*21.2*	*-0.220*	*0.265*
	A. yunnanensis	*1,898*	*31.9*	*47.0*	*8.0*	*13.1*	*78.9*	*21.1*	*-0.192*	*0.240*
	G. genistae	*1,910*	*32.4*	*47.5*	*7.3*	*12.8*	*79.9*	*20.1*	*-0.189*	*0.276*
	T. longivalvulatus	*1,370*	*31.1*	*45.8*	*8.4*	*14.7*	*76.9*	*23.1*	*-0.191*	*0.274*
Full genome	*A. lineatus*	*16,218*	*45.3*	*31.1*	*14.4*	*9.2*	*76.4*	*23.6*	*0.186*	*-0.218*
	A. yunnanensis	*14,755*	*45.3*	*31.0*	*14.5*	*9.2*	*76.3*	*23.7*	*0.186*	*-0.220*
	G. genistae	*15,829*	*43.0*	*34.0*	*12.0*	*10.9*	*77.0*	*22.9*	*0.117*	*-0.045*
	T. longivalvulatus	*15,325*	*44.2*	*32.7*	*13.6*	*9.6*	*76.9*	*23.2*	*0.150*	*-0.169*
