# Supplementary material for: Characterization, Comparison of Four New Mitogenomes of Centrotinae (Hemiptera: Membracidae) and Phylogenetic Implications Supports New Synonymy
Source: Life (Basel). 2022 Jan 3;12(1):61. doi: 10.3390/life12010061 (PMC8777817; doi:10.3390/life12010061)
Supplement: Supplementary file 1 [file life-12-00061-s001.zip › Supplementary Table S4.pdf]

Table S4. Organization of the mitogenome of *A. lineatus*.

Name	Location		Size(bp)	Intergenic nucleotides	Codon		Strand
	From	To			Start	Stop	
trnI	1	66	66				+
trnQ	64	131	68	-3			-
trnM	131	196	66	-1			+
nad2	197	1,156	960		ATG	TAG	+
trnW	1,156	1,219	64	-1			+
trnC	1,219	1,282	64	-1			-
trnY	1,283	1,346	64				-
cox1	1,345	2,878	1,534	-2	ATG	T	+
tRNA-Leu2	2,879	2,944	66				+
cox2	2,945	3,620	676		ATA	T	+
trnK	3,618	3,687	70	-3			+
trnD	3,688	3,751	64				+
atp8	3,752	3,904	153		ATA	TAA	+
atp6	3,898	4,548	651	-7	ATG	TAA	+
cox3	4,549	5,328	780		ATG	TAA	+
trnG	5,329	5,388	60				+
nad3	5,392	5,742	351	3	ATA	TAA	+
trnA	5,741	5,803	63	-2			+
trnR	5,805	5,866	62	1			+
trnN	5,867	5,929	63				+
tRNA-Ser1	5,929	5,994	66	-1			+
trnE	5,994	6,058	65	-1			+
trnF	6,058	6,120	63	-1			-

Table S4. *Cont*.

nad5	6,121	7,783	1,663		ATT	T	-
trnH	7,787	7,847	61	3			-
nad4	7,850	9,160	1,311	2	ATG	TAA	-
nad4L	9,154	9,429	276	-7	ATG	TAA	-
trnT	9,432	9,493	62	2			+
trnP	9,494	9,556	63				-
nad6	9,559	10,044	486	2	ATA	TAA	+
cytb	10,037	11,170	1,134	-8	ATG	TAG	+
trnS2	11,169	11,232	64	-2			+
nad1	11,232	12,167	936	-1	ATA	TAA	-
trnL1	12,165	12,230	66	-3			-
rrnL	12,231	13,392	1,162				-
trnV	13,393	13,455	63				-
rrnS	13,455	14,278	824	-1			-
CR	14,279	16,218	1,940				+
