# Supplementary material for: Characterization, Comparison of Four New Mitogenomes of Centrotinae (Hemiptera: Membracidae) and Phylogenetic Implications Supports New Synonymy
Source: Life (Basel). 2022 Jan 3;12(1):61. doi: 10.3390/life12010061 (PMC8777817; doi:10.3390/life12010061)
Supplement: Supplementary file 1 [file life-12-00061-s001.zip › Supplementary Table S5.pdf]

Table S5. Organization of the mitogenome of *A. yunnanensis*.

Name	Location		Size(bp)	Intergenic nucleotides	Codon		Strand
	From	To			Start	Stop	
trnI	1	66	66				+
trnQ	64	131	68	-3			-
trnM	131	197	67	-1			+
nad2	198	1,157	960		ATG	TAG	+
trnW	1,157	1,218	62	-1			+
trnC	1,218	1,282	65	-1			-
trnY	1,283	1,346	64				-
cox1	1,345	2,878	1,534	-2	ATG	T	+
trnL2	2,879	2,944	66				+
cox2	2,945	3,617	673		ATT	T	+
trnK	3,618	3,687	70				+
trnD	3,688	3,750	63				+
atp8	3,751	3,903	153		ATA	TAA	+
atp6	3,897	4,547	651	-7	ATG	TAA	+
cox3	4,548	5,327	780		ATG	TAA	+
trnG	5,327	5,387	61	-1			+
nad3	5,388	5,741	354		ATA	TAA	+
trnA	5,740	5,802	63	-2			+
trnR	5,804	5,866	63	1			+
trnN	5,866	5,930	65	-1			+
trnS1	5,930	5,995	66	-1			+
trnE	5,995	6,057	63	-1			+
trnF	6,057	6,120	64	-1			-

Table S5. *Cont.*

nad5	6,121	7,786	1,666		TTG	T	-
trnH	7,787	7,847	61				-
nad4	7,850	9,154	1,305	2	ATG	TAA	-
nad4L	9,148	9,423	276	-7	ATG	TAA	-
trnT	9,426	9,486	61	2			+
trnP	9,487	9,549	63				-
nad6	9,552	10,037	486	2	ATA	TAA	+
cytb	10,030	11,163	1,134	-8	ATG	TAG	+
trnS2	11,162	11,225	64	-2			+
nad1	11,225	12,157	933	-1	ATT	TAA	-
trnL1	12,158	12,224	67				-
rrnL	12,225	13,386	1,162				-
trnV	13,387	13,449	63				-
rrnS	13,450	14,185	736				-
CR	14,186	14,755	570				+
