# Supplementary material for: Characterization, Comparison of Four New Mitogenomes of Centrotinae (Hemiptera: Membracidae) and Phylogenetic Implications Supports New Synonymy
Source: Life (Basel). 2022 Jan 3;12(1):61. doi: 10.3390/life12010061 (PMC8777817; doi:10.3390/life12010061)
Supplement: Supplementary file 1 [file life-12-00061-s001.zip › Supplementary Table S6.pdf]

Table S6. Organization of the mitogenome of *G. genistae.*

Name	Location		Size(bp)	Intergenic nucleotides	Codon		Strand
	From	To			Start	Stop	
trnI	1	64	64				+
trnQ	62	129	68	-3			-
trnM	129	195	67	-1			+
nad2	196	1,158	963		ATT	TAG	+
trnW	1,157	1,218	62	-2			+
trnC	1,219	1,278	60				-
trnY	1,279	1,341	63				-
cox1	1,340	2,873	1,534	-2	ATG	T	+
trnL2	2,874	2,939	66				+
cox2	2,940	3,621	682		ATA	T	+
trnK	3,619	3,689	71	-3			+
trnD	3,690	3,751	62				+
atp8	3,752	3,904	153		ATA	TAA	+
atp6	3,898	4,548	651	-7	ATG	TAA	+
cox3	4,549	5,328	780		ATG	TAA	+
trnG	5,327	5,386	60	-2			+
nad3	5,391	5,741	351	4	ATA	TAA	+
trnA	5,739	5,798	60	-3			+
trnR	5,800	5,862	63	1			+
trnN	5,861	5,927	67	-2			+
trnS1	5,927	5,992	66	-1			+
trnE	5,992	6,053	62	-1			+
trnF	6,053	6,115	63	-1			-

Table S6. *Cont.*

nad5	6,117	7,779	1,663	1	ATA	T	-
trnH	7,782	7,842	61	2			-
nad4	7,845	9,149	1,305	2	ATG	TAA	-
nad4L	9,143	9,418	276	-7	ATG	TAA	-
trnT	9,420	9,478	59	1			+
trnP	9,479	9,544	66				-
nad6	9,548	10,039	492	3	ATT	TAA	+
cytb	10,026	11,162	1,137	-14	ATG	TAG	+
trnS2	11,160	11,224	65	-3			+
nad1	11,219	12,154	936	-6	ATT	TAA	-
trnL1	12,160	12,224	65	5			-
rrnL	12,225	13,395	1,171				-
trnV	13,396	13,457	62				-
rrnS	13,458	14,196	739				-
CR	14,197	15,829	1,633				+
