# Supplementary material for: Characterization, Comparison of Four New Mitogenomes of Centrotinae (Hemiptera: Membracidae) and Phylogenetic Implications Supports New Synonymy
Source: Life (Basel). 2022 Jan 3;12(1):61. doi: 10.3390/life12010061 (PMC8777817; doi:10.3390/life12010061)
Supplement: Supplementary file 1 [file life-12-00061-s001.zip › Supplementary Table S7.pdf]

Table S7. Organization of the mitogenome of *T. longivalvulatus*.

Name	Location		Size(bp)	Intergenic nucleotides	Codon		Strand
	From	To			Start	Stop	
trnI	1	62	62				+
trnQ	60	128	69	-3			-
trnM	156	221	66	27			+
nad2	222	1,190	969		ATT	TAA	+
trnW	1,181	1,241	61	-10			+
trnC	1,242	1,301	60				-
trnY	1,303	1,364	62	1			-
cox1	1,363	2,896	1,534	-2	ATG	T	+
trnL2	2,897	2,962	66				+
cox2	2,963	3,641	679		ATA	T	+
trnK	3,642	3,712	71				+
trnD	3,713	3,774	62				+
atp8	3,775	3,927	153		ATA	TAA	+
atp6	3,921	4,568	648	-7	ATG	TAA	+
cox3	4,569	5,346	778		ATG	T	+
trnG	5,347	5,407	61				+
nad3	5,411	5,761	351	3	ATT	TAA	+
trnA	5,760	5,822	63	-2			+
trnR	5,826	5,885	60	3			+
trnN	5,885	5,949	65	-1			+
trnS1	5,949	6,012	64	-1			+
trnE	6,012	6,074	63	-1			+
trnF	6,075	6,136	62				-

Table S7. *Cont.*

nad5	6,137	7,802	1,666		TTG	T	-
trnH	7,803	7,864	62				-
nad4	7,867	9,171	1,305	2	ATG	TAA	-
nad4L	9,165	9,440	276	-7	ATG	TAA	-
trnT	9,443	9,503	61	2			+
trnP	9,504	9,565	62				-
nad6	9,568	10,053	486	2	ATA	TAA	+
cytb	10,046	11,182	1,137	-8	ATG	TAA	+
trnS2	11,184	11,247	64	1			+
nad1	11,247	12,179	933	-1	ATT	TAA	-
trnL1	12,180	12,244	65				-
rrnL	12,272	13,426	1,154				-
trnV	13,427	13,488	62				-
rrnS	13,491	14,226	736	2			-
CR	14,227	15,325	1,099				+
